# Carotenoids of *Capsicum* Fruits: Pigment Profile and Health-Promoting Functional Attributes

**DOI:** 10.3390/antiox8100469

**Published:** 2019-10-09

**Authors:** Norazian Mohd Hassan, Nurul Asyiqin Yusof, Amirah Fareeza Yahaya, Nurul Nasyitah Mohd Rozali, Rashidi Othman

**Affiliations:** 1 Department of Pharmaceutical Chemistry, Kulliyyah of Pharmacy, International Islamic University Malaysia, Kuantan 25200, Malaysia; amirahfareeza.yahaya@gmail.com (A.F.Y.); nurul_2293@yahoo.com (N.N.M.R.); 2 Department of Basic Medical Sciences, Kulliyyah of Pharmacy, International Islamic University Malaysia, Kuantan 25200, Malaysia; drnurul@iium.edu.my; 3 International Institute for Halal Research and Training (INHART), Herbarium Unit, Department of Landscape Architecture, Kulliyyah of Architecture and Environment Design, International Islamic University Malaysia, Kuala Lumpur 53100, Malaysia; rashidi@iium.edu.my; 4 Department of Landscape Architecture, Kulliyyah of Architecture and Environmental Design, International Islamic University Malaysia, Kuala Lumpur 53100, Malaysia

**Keywords:** *Capsicum*, fruit, carotenoids, functional attributes, health promotion

## Abstract

Pepper of the *Capsicum* species is a common ingredient in various food preparations by different cultures worldwide. The *Capsicum* is recognised by its five main domesticated species, namely *Capsicum annuum, C. baccatum, C. chinense, C. frutescens* and *C. pubescens*. The genetic diversity in *Capsicum* offers fruits in wide ranges of morphology and carotenoid profile. Carotenoids enhance the value of pepper from a nutritional standpoint, despite being commonly prized for the pharmacologically active pungent capsaicinoids. Carotenoids of pepper comprise mainly of the unique, powerful and highly stable capsanthin and capsoroubin, together with β-carotene, β-cryptoxanthin, lutein, zeaxanthin, antheraxanthin and violaxanthin. These carotenoids are present at diverse profile and varying levels, biosynthetically connected to the fruit maturity stages. This review describes the health-promoting functional attributes of the carotenoids that are mainly associated with their excellent role as lipophilic antioxidants. *Capsicum* as a great source of carotenoids is discussed in the aspects of main domesticated species, biosynthesis, pigment profile, antioxidant activity and safety. Findings from a number of in vitro, in vivo and clinical studies provided appreciable evidence on the protective effects of pepper’s carotenoids against degenerative diseases. Hence, pepper with its functional carotenoids might be recommended in health-promoting and disease preventing strategies.

## 1. Introduction

The fruit of the *Capsicum* species, which is also known as pepper, is one of the common ingredients used in various cuisines worldwide for its unique flavours, either spicy, hot, sweet or sometimes sour. These unique tastes made some of the general population, especially those who are living around Asia, the Mediterranean and Southern America, crave to have them in their daily diets either in raw, cooked or processed products [1]. However, there are also those who hate pepper due to the unbearable spiciness that happens to be a characteristic feature of some of the *Capsicum* species, varieties and cultivars for the pungent components, capsaicinoids [2]. *Capsicum* species are also used traditionally as medicine by the Asian cultures, especially the Chinese and Indian together with the Native Americans [3].

Pepper is a carotenoids-rich non-leafy vegetable. Carotenoids are tetraterpenoids comprising of a central polyenic chain of nine conjugated double-bonds and a variety of end groups as the chromophore that confers each pigment’s properties [4]. The carotenes are the hydrocarbon carotenoids whereas the xanthophylls are the oxygenated derivatives that usually occur in esterified forms with fatty acids [5,6]. Taxonomically, pepper is ranked under the genus *Capsicum* of the Solanaceae family. It originates from Central and Southern America. The fruits may vary in terms of shape, size, colour, taste and chemical composition due to their genetic diversity and the remarkable changes in physiology and biochemistry during ripening [7,8]. Paprika and its oleoresin are pepper products rich in capsanthin and mixed carotenoids, mainly β-carotene, β-cryptoxanthin, zeaxanthin and capsorubin of *C. annuum*. Both are natural colouring with less side effects, are non-carcinogenic and possess known biological functionalities associated with disease prevention. In addition, they are safe alternatives to synthetic colouring agents which impart red colour to nutraceutical, cosmeceutical and pharmaceutical products [9,10,11].

Nevertheless, most of the general population are still oblivious to *Capsicum* as a carotenoids-rich source, especially for the predominant capsanthin, and its health-promoting functional attributes. In fact, the feeling of fear of cancer risk due to *Capsicum* intake rises. A meta-analysis of evidence from case-control studies on the association between chili pepper-containing spicy food intake and cancer risk has suggested an increased risk of having gastric cancer for those with high pepper intake, presumably due to the carcinogenic dose-effects of capsaicin [12]. Thus, this review describes the health-promoting functional attributes of the *Capsicum* focusing on its main carotenoids, namely, capsanthin, capsorubin, β-carotene, β-cryptoxanthin, lutein, zeaxanthin, antheraxanthin and violaxanthin. In addition, the aspects of main domesticated species, biosynthesis, carotenoid profile, antioxidant activity and safety are discussed with regard to *Capsicum* as a great source of carotenoids.

## 2. The *Capsicum* Species 

**Figure 1:**
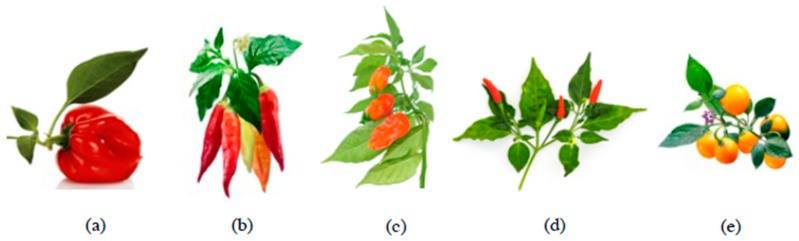
*Capsicum* fruits: (**a**) *C*. *annuum* (sweet bell pepper), (**b**) *C*. *baccatum* (aji pepper), (**c**) *C*. *chinense* (bhut jolokia pepper), (**d**) *C*. *frutescens* (bird’s eye chilli) and (**e**) *C*. *pubescens* (rocoto pepper).

The genus *Capsicum* consists of about thirty-seven species comprising of wild, semi-domesticated and the five well-known domesticated species, namely, *C. annuum* L., *C. baccatum* L., *C. chinense* Jacq., *C. frutescens* L. and *C. pubescens* Ruiz and Pav (Figure 1), with more than 200 varieties [13]. *Capsicum* species are native to the Central and Southern America, where Brazil is the centre for most wild peppers while Peru and Bolivia are the primary centre of cultivated *Capsicum* diversity [14]. *C. annuum,* of which domestication and genetic diversity is centred in Mexico, is the most widely grown and economically important species worldwide [3,15]. *C. chinense* is the most widespread species in tropical America, whereas *C. frutescens* is being naturalized and has become popular in Asia and Africa. Meanwhile, *C. baccatum* and *C. pubescens* are mostly distributed within South America and the Andean region, where *C. baccatum,* or as it is locally known as aji, is the more preferred domesticated species [13]. *C. annuum*, *C. chinense* and *C. frutescens* are phylogenetically close sister species and are sometimes referred to as “*annuum*-*chinense*-*frutescens*” complex for their overlapping morphological features [16]. Based on flower morphology, anther colour is the important character used in the identification of *Capsicum* species, by which *C. baccatum* accessions possess yellow anther while those of *C. annuum* are characterized by their purple anthers and the occurrence of one solitary flower per axil. *C. annuum, C. chinense* and *C. frutescens* have flowers with greenish to white coloured petals and yellow seeds [14,17]. *C. pubescens* is characterized by its flowers with distinctive purple corolla and black rough seeds, while *C. baccatum* is characterized by its white corolla with yellow-green spots and yellow seeds [14].

The various cultivars of *C. annuum* are classified mainly based on their fruit morphological characters, like shape, including round/oval and long/thin, size, pericarp surface and sacrocarp thickness, into, for instance, Bell, Pimento, Ancho, Anaheim, Jalapeno, Cherry, Long Wax and Sishi [18]. In mature state, the fruits of most cultivars are red in colour, flattened in shape and have several locules [17]. The quality of *Capsicum* fruits is determined, among others, by its colour, pungency and flavour attributes, which are imparted by its pigments, especially carotenoids mixture, pungent capsicinoids and volatile compounds profiles, respectively [19,20]. Based on pungency, pepper can be grouped into hot and non-hot types, by which, to date, ‘Trinidad moruga scorpion’ pepper, a *C. chinense* cultivar, is regarded as the world’s hottest chilli pepper [21], whereas ‘Pimenta’ is the non-pungent highly aromatic cultivar [20]. A rich inter- and intra-specific genetic variation has led to the emergence of a high number of varieties and cultivars with a great advantage of yielding improved quality fruits [22,23].

## 3. Carotenoids of *Capsicum* Species

**Figure 2:**
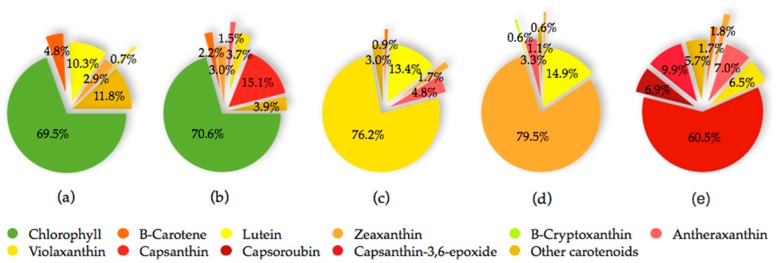
Composition of the main pigments in selected variety (var.)/cultivars (cv.) of *C*. *annuum* fruits at different maturity stages (**a**) green (var. *lycopersiciforme rubrum*), (**b**) brown (cv. Marajá), (**c**) yellow (cv. Vílez), (**d**) orange (cv. Mazzona), and (**e**) red (cv. Ferari) [25,30,31,32].

**Figure 3:**
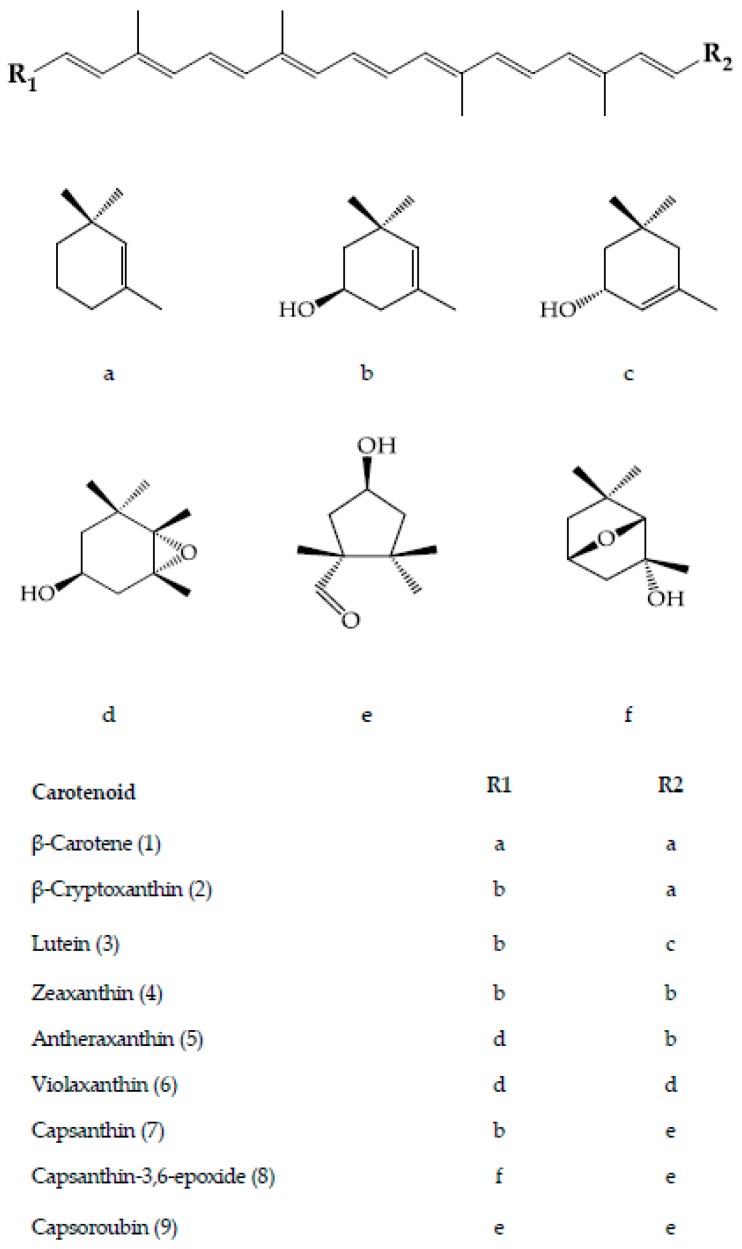
Main carotenoids of different fruit colours of *Capsicum* species [9,33,34,35,36].

The fruit of the *Capsicum* species is recognized as a carotenoids-rich non-leafy vegetable. The diverse carotenoids are present in the sacrocarps and can be grouped into yellow, orange and red carotenoids, which impart pale yellow to dark red colours to the fruits [24]. The carotenoids develop and accumulate rapidly as the fruit ripens. At the beginning, the fruit is green in colour, which is full of chloroplast containing approximately 68% chlorophylls, whereas carotenoids at 32% is at the lowest level [25]. At this stage, the typical chloroplast carotenoids like lutein, violaxanthin, neoxanthin and β-carotene co-exist with and are masked by chlorophylls [26]. As the fruit ripens, the chromoplast carotenoids synthesis occurs through transformation of the existing chloroplast carotenoids and de novo synthesis. Throughout the ripening process, the chloroplast is differentiated into chromoplast containing mixed carotenoids which contribute collectively to different fruit colours from green to brown then to yellow, orange, red and/or dark red at the final maturity stage, dependent on the cultivars [25,27,28,29]. Thus, the different colours of pepper fruit can be characterized by the pigment profile, as summarized and depicted in Figure 2 for the main carotenoids, with the structures as depicted in Figure 3.

The red pepper is unique for its high xanthophylls content and composition. It shows the most diverse carotenoid profile consists mainly of the yellow carotenoids namely β-carotene, violaxanthin, antheraxanthin, zeaxanthin, and the characteristic intense red ketocarotenoids, which are capsanthin, capsorubin and capsanthin-5,6-epoxide. The main carotenoids of a fully ripe yellow/orange pepper include lutein, β-carotene, violaxanthin, antheraxanthin, zeaxanthin and β-cryptoxanthin [28,30,37,38]. The most complex carotenoids profile is observed at the intermediate ripening stage in which pepper may consists of up to sixty four different free, partially and fully esterified carotenoids. At brown intermediate stage, the chlorophylls remain as the profound pigment while capsanthin is the major carotenoid of red intermediate stage [31].

Besides maturity stage at harvest, the variation in composition and relative content of carotenoids of the *Capsicum* species is influenced mainly by the differences in genotypes, agro-climatic conditions, post-harvest handling, processing and preparation [4,39,40,41]. A considerable variation in carotenoid profile among species and cultivars of *Capsicum* is associated with the difference in genotypes which determine their specific carotenoids biosynthetic enzymes [37]. Generally, the orange colour of most of the orange pepper cultivars is due to the abundance in β-carotene, with the exception of a few intense orange-fruited cultivars which resulted from a mixture of red pigment, capsanthin and yellow xanthophylls, namely β-cryptoxanthin and violaxanthin [38,40,42]. The absence and inactivation of capsanthin-capsoroubin synthase (CCS) enzyme in certain yellow/orange cultivars has blocked the red pigments synthesis and let their precursors, such as violaxanthin, accumulate as the major carotenoid at the fully ripe stage [30].

Analysis of 51 pepper genotypes grouped by species showed mean total carotenoids of 23.21 ± 23.55, 28.47 ± 19.34, 34.97 ± 34.94 mg β-carotene/100 g fresh weight respectively, for *C. chinense*, *C. annuum* and *C. baccatum,* among which the red accessions P269, P280 and P179 are those with the highest content [43]. The *Capsicum* sp. of different genotypes from *C. annuum*, *C. chinense* and *C. baccatum* var. *umbilicatum* demonstrated a variation in carotenoid profile and concentration that reflected on various fruit colours [32,44]. Variable levels of main carotenoids in selected collections of sweet and hot *Capsicum* are shown in Table 1. In *C*. *annuum*, β-carotene accumulation is favoured by grafting, as shown by 5.8-fold greater content in Orangela/Terrano than other cultivar/rootstock combinations and varieties that are grown under net shading and harvested in September [45]. The ground paprika of *C. annuum* cv. Kalocsai V2 (hot pepper) and *C. annuum* cv. Szegedi 80 (sweet pepper) from several production sites in Croatia, Serbia and Hungary were greatly varied in their capsanthin and capsoroubin content. The highest content was found in those grown in Croatia, which showed the influence of geographical origin on carotenoid metabolism [46]. Furthermore, reduced light stress in a shaded greenhouse promoted carotenoids accumulation in three orange-fruited *C. annuum* cultivars, namely, Fogo, NuMex Sunset and Orange Grande. Lutein, zeaxanthin and violaxanthin were the major carotenoids that presented at the highest concentrations of 41.29, 34.50 and 83.24 µg/g fresh weight respectively, in Fogo [47]. 

The main carotenoids of *C*. *annuum* include capsanthin, capsorubin, β-carotene, zeaxanthin, violaxanthin, lutein and antheraxanthin, which vary in concentration at different stages of fruit maturity [25]. For red *C*. *pubescens*, capsanthin, antheraxanthin, mutatoxanthin, violaxanthin and β-carotene are the five main carotenoids, whereas in the red *C*. *baccatum*, the similar carotenoids are present, however, with additional capsanthin-5,6-epoxide at a higher level [30]. Lutein and its isomer, zeaxanthin, differ in the position of the double bond between the unsaturated β-ionone ring and the ε-ionone ring. In *Capsicum* species, lutein is abundantly present (approximately 30%) at the green stage and exclusively in habanero white cultivar of *C*. *chinense* (48.3%) [25,37]. It is the primary carotenoid in yellow-fruited varieties besides β-carotene. During ripening, its level decreases to the least in red-fruited cultivars [29], whereas it remains in those of yellow/orange [1,30]. Orange cultivars of *C*. *annuum* are the best pepper source of zeaxanthin, with total content covering over 80%, ranging between 85.06 ± 23.37 and 151.39 ± 5.94 mg/100 g dry weight [32].

β-Carotene is a ubiquitous hydrocarbon carotenoid in plant tissues including pepper and is naturally present in a stable all-trans isomer. It is highly valued for its nutritional and colour properties, and has a variety of applications in food, pharmaceuticals and cosmetics [48]. Generally, its content is higher in fresh hot pepper than that of the sweet cultivars [49]. β-Carotene concentration is proven to directly correlate to the total carotenoid content of a genotype as it is the precursor for the predominant orange and red carotenoids of pepper [33]. Contrary to β-carotene, its hydroxy derivative, β-cryptoxanthin, presents in high concentration in limited food sources. In pepper, its content is the highest in dried fruit, while paprika oleoresin is the best source for bioavailable β-cryptoxanthin [34]. Both β-carotene and β-cryptoxanthin possess orange colour due to the same chromophore length with maximum absorption at the wavelength of 450 nm. The carotenoids are present in almost all pepper cultivars at cultivar-specific abundance [38] and are the main carotenoids of the orange-fruited *Capsicum* cultivars [42,45]. Besides, β-carotene also presents consistently at an approximate ratio of 1:10 to capsanthin in mature red fruits in most cultivars of different groups of *C*. *annuum* [18]. The content may reach up to four folds and more in fresh fruit of red cultivars than in yellow-/orange-fruited cultivars [33].

Antheraxanthin and violaxanthin are the epoxy-xanthophylls with one and two epoxy groups, respectively. Violaxanthin, *cis*-violaxanthin and antheraxanthin are the main carotenoids presented in the less complex carotenoids profile of the fully ripe yellow-/orange-fruited genotypes of *C. annuum* and the Andean peppers, *C. baccatum* and *C. pubescens,* in which all red carotenoids were absent. The yellow-/orange-fruited *C. annuum* and *C. baccatum* were excellent sources for violaxanthin (approximately 68% of the total carotenoids), corresponding to ten and seven times higher respectively, than that of the red-fruited genotypes. In addition, violaxanthin was approximately eighteen and eight times higher than β-carotene respectively, in *C. baccatum* and *C. pubescens* yellow-/orange-fruited, whereas only about two-fold in that of the red-fruited ones. Its *cis* isomer and antheraxanthin presented at 5% to 14% [30].

Capsanthin and capsoroubin are the predominant red xanthophylls of the red ripe pepper. Both are κ-ring ended keto xanthophylls with a distinctive characteristic of an eleven conjugated double-bond system consisting of a central polyene chain with one and two acylcyclopentanol end group(s) for capsanthin and capsoroubin, respectively. The unique colour of ripe red peppers is due to the chemistry of the red light-absorbing chromophore of polyene chain and the efficient green light-absorbing keto group which imparts the brilliant red colour and red-orange hue, respectively [9,39]. Generally, the dark red coloured fruit presents the highest total carotenoids content compared with those with lighter colour and non-red pepper with decreasing order of dark red > light red > orange > yellow, while most of the white type contains no carotenoids. The amount in red pepper is approximately four to five times higher than in green pepper (respectively, 362 ± 7.8 and 62.7 ± 5.5 mg/100g fresh weight) [50]. The level of capsanthin is significantly increased during the ripening of red peppers. It is the principal pigment which accounts for almost 80% of the total carotenoids, ranging from 230 to 848 µg/100 g fresh weight in *C. annuum*, *C. baccatum* and *C. pubescens* [1,29,30]. Its content declined proportionally with colour that ranges from dark red, red, light red, orange and pale yellow-orange [29]. Capsorubin is only detected at the final stage of fruit maturity, specifically in matured brown cultivar and the deep red stage of red cultivar of *C. annuum* at low concentrations (3.86 and 3.17 mg/100 g dry weight, respectively) [25].

The red pepper is also a fruit rich in xanthophyll esters as most of zeaxanthin, antheraxanthin, β-cryptoxanthin, capsanthin and capsorubin are predominantly present in their fatty acids’ mono- and diesterified forms. The esterification and de novo synthesis of the xanthophylls occurs simultaneously during ripening which increase the carotenoids’ liposolubility and stability [37,51]. As the fruit ripens, an account of about 70% to 80% of total capsanthin is esterified with fatty acids which facilitate their incorporation into the membrane structure. Capsanthin predominantly presents as esters (approximately 98%) with lauric, palmitic and myristic acid, which showed higher bioaccessibility and stability than other carotenoids of dried red pepper. This is in contrast with green pepper, in which most of the carotenoids, such as lutein, are present as free carotenoids possessing less stable and less bioaccessible properties [35,40]. A series of metabolic reactions such as oxidative cleavage and isomerization could also occur at the late ripening stage which results in a considerable complex carotenoid profile in addition to several minor carotenoids [28,35,52]. The carotenoids of pepper undergo eccentric oxidative cleavages yielding apocarotenoids such as apozeaxanthinals and apocapsorubinals from zeaxanthin and capsorubin respectively [53,54], which also possess potential preventive roles against degenerative diseases [5].

In *C*. *annuum*, several minor carotenoids are specifically present at different maturity stages. The carotenoids of the green fruit include 5,6-diepilatoxanthin, luteoxanthin 1*c* and luteoxanthin 2*c* with concentrations of 1.74, 3.62 and 1.97 mg/100 g dry weight (DW), respectively. 6-Epikarpoxanthin, neoxanthin, capsanthin-3,6-epoxide, α-cryptoxanthin and α-carotene are found in the pale green pepper at the concentrations of 4.50, 6.97, 0.42, 0.66 and 0.79 mg/100 g DW, respectively. As for the brownish pepper, 5,6-diepicapsokarpoxanthin, 5,6-diepikarpoxanthin and *cis*-β-carotene are found at concentrations of 0.36, 4.78 and 1.39 mg/100 g DW, respectively [25]. Meanwhile, capsanthin-5,6-epoxide, cucurbitaxanthin B, cucurbitachrome, 8*S*-mutoxanthin, 8*R*-mutoxanthin, cucurbitaxanthin A, 9/9′-*cis*-capsanthin, 13/13′-*cis*-capsanthin, nigroxanthin, crytocapcapsin, *cis*-cryptoxanthin, 3′-deoxycapsanthin and 3,4-dehydroxy-3′-deoxycapsanthin are those of the deep red fruit [25,55].

## 4. Biosynthesis of Carotenoids in *Capsicum* Species 

**Figure 4:**
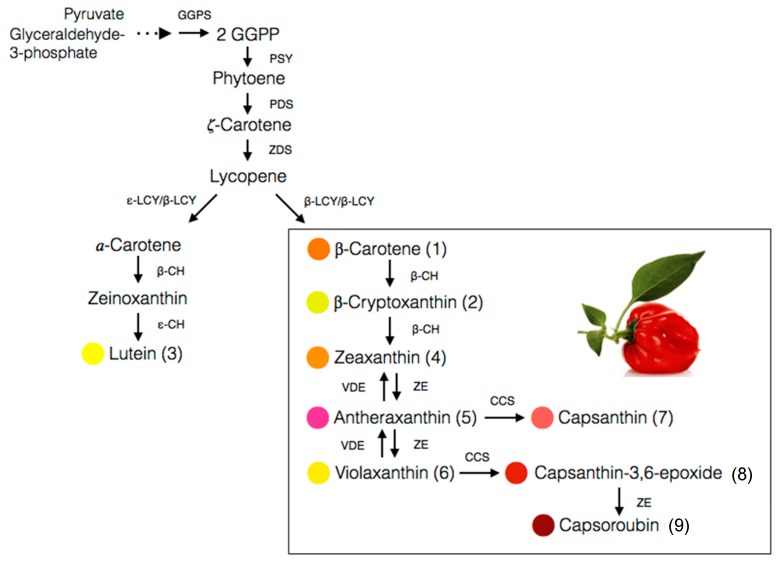
Biosynthetic pathways of main carotenoids in the red-fruited *Capsicum* species. GGPP, geranylgeranyl pyrophosphate; GGPS, geranylgeranyl pyrophosphate synthase; PSY, phytoene synthase; PDS, phytoene desaturase; ZDS, ζ-carotene desaturase; β-LCY, lycopene-β-cyclase; ε-LCY, lycopene-ε-cyclase; β-CH, β-carotene hydroxylase; ε-CH, ε-carotene hydroxylase; ZE, zeaxanthin epoxidase; VDE, violaxanthin epoxidase; CCS, capsanthin-capsoroubin synthase [29,55].

The carotenoid biosynthesis in pepper is an observable process of gradual changes in colour during fruit ripening from green to yellow, orange, and finally red, depending on the cultivars. It is actually the process of chlorophylls degradation and carotenoids synthesis as the chloroplasts differentiate into chromoplasts [27]. About 95% of the carotenoids are accumulated in the fibrils of the chromoplast while the rest, 5%, are floating freely in the organelle [28]. The biosynthesis pathway for the main carotenoids of the *Capsicum* species, particularly the red-fruited types, is depicted in Figure 4. The starting compound is known as geranylgeranyl pyrophosphate (GGPP) which is converted to phytoene in a reaction catalysed by phytoene synthase (PSY). Next, phytoene desaturase (PDS) and ζ-carotene desaturase (ZDS) catalyse desaturation of phytoene into lycopene. The pivotal point in the pathway of carotenoids biosynthesis in pepper is the cyclization of both ends of lycopene to form β-carotene catalysed by the chromoplast membrane-derived enzyme, lycopene β-cyclase (LCYB). Hydroxylation of α-carotene at β- and ε-ring give rise to lutein, whereas zeaxanthin is biosynthesized from β-carotene through a two-step reaction, with β-carotene hydroxylase (BCH) enzyme and CYP97A as the catalysts [56].

Antheraxanthin is converted from zeaxanthin in the presence of the zeaxanthin epoxidase (ZEP) enzyme, which attacks one of its β-ring to produce an epoxide ring. Further attacks by ZEP on the β-ring of antheraxanthin produced violaxanthin with two epoxide rings [28]. It is an interconversion process closely regulated by xanthophyll cycle, which rapidly occurs in chromoplast and induces by changes in light intensity [57]. Capsanthin and capsorubin biosynthesis is catalysed by capsanthin-capsorubin synthase (CCS) from their 5,6-epoxycarotenoid predecessors, antheraxanthin and violaxanthin, respectively [39,58]. CCS is present at the highest concentration in the brown matured or red stages of ripening in *C. annuum* [28] as the CCS gene is highly expressed during the process [59].

## 5. Antioxidant Activities of Pepper’s Carotenoids

Carotenoids are among the most significant antioxidants of pepper besides phenolics and flavonoids which act synergistically as efficient free radical scavengers [7,45,60]. The free radical scavenging ability of carotenoids comes from its extended linear system of conjugated double bonds, which allows resonance to occur and maintain the stability of its structure [61]. Carotenoid terminated the free radicals by transferring electrons, forming adduct (joined to the free radical) or donating hydrogen to form relatively stable carotenoid radicals. The number of conjugated double bonds in carotenoids’ structures significantly contributes to their role as lipophilic antioxidants. The liphophilicity is also influenced by the presence and number of functional groups, such as carbonyl and hydroxyl groups [62].

The antioxidant activity of pepper increases with advanced stages of ripening as more carotenoids are synthesized [63]. In different *C. annuum* cultivars, a gradual increment, up to six-fold, of β-carotene with a strong positive correlation to antioxidant activity was observed from the green mature to the red ripe stage [7]. In addition, the carotenoid-rich fractions of ripened fruits of *C. annuum* cultivars with the highest content of β-carotene and capsanthin exhibited stronger DPPH radical scavenging and reducing power activities than those of the unripe stages [64]. Nevertheless, the antioxidant activity of a carotenoid extract is determined by both its carotenoids’ constituent and ratio. For instance, extracts containing the same carotenoids constituent from guajillo, pasilla and ancho pepper with total carotenoid content of 3.4, 2.9 and 1.4 mg/g dry weight respectively, exhibited different strength of antioxidant activity due to the difference in their carotenoids’ ratio [3]. Besides, a significant (*p* < 0.05) synergistic 2,2’-azino-bis(3-ethylbenzothiazoline-6-sulfonic acid)diammonium salt (ABTS) radicals scavenging activity was observed in a 40 µM mixture of β-carotene and capsanthin at the ratios of 1:1 and 1:9, while antagonistic combination effect (*p* < 0.05) was observed at the ratio of 9:1 [65]. The carotenoids’-associated antioxidant properties of pepper is also influenced by the thermal treatments during postharvest handling, processing and preparation, by which at certain conditions, the properties might be enhanced or declined [31,41,66,67,68].

The most powerful antioxidant carotenoids of pepper are capsanthin and capsorubin, which are the characteristic xanthophylls of the red fruits [69]. Although capsanthin and capsorubin have a very low bioavailability compared with β-carotene [70], both possess stronger antioxidant activity which resulted from their conjugated keto extended polyene chain compared with that of the non-extended chain in β-carotene [71]. Antioxidant inhibitory effect of carotenoids of *C. annuum* against peroxyl radical-dependent lipid peroxidation showed the strength of activity in the decreasing order of capsorubin > capsanthin 3,6-epoxide > capsanthin > cycloviolaxanthin > β-carotene, which revealed the red xanthophylls as stronger lipid peroxidation inhibitors than β-carotene [72]. Capsorubin also possessed superior singlet oxygen quencher to cucurbitaxanthin A, capsanthin and the positive control, astaxanthin [36]. The excellent antioxidant activity of capsorubin is exhibited by its two conjugated keto groups enhanced polyene chain [58]. Meanwhile, the antioxidant activity and oxidative degradation of capsanthin and capsoroubin esters have no influences by their fatty acid moieties, since only the extended polyene chain plays the role for the activity [70,73].

## 6. Health-Promoting Functional Attributes 

Consumption of hot red chili pepper has been associated with a significant decrease in mortality, as revealed through two large population-based cohort studies after adjustment of the risk factors involving China and US populations [74,75]. During a median follow-up of 7.2 years (interquartile range 1.84 years; total person years 3,500,004), participants who consumed spicy food six or seven days a week showed a 14% relative risk reduction in total mortality and inverse associations for deaths due to cancer, ischemic heart diseases, and respiratory diseases [74]. A corroborative finding of a 13% reduction in the instantaneous hazard of death was observed during a median follow-up of 18.9 years representing 273,877 US adults that suggested the protective effect of chili pepper may partly be contributed by its antioxidants, specifically the carotenoids [75].

Antioxidants are regarded as the indicator for overall health benefits of pepper, exerted, among others, by its rich xanthophylls, which are anti-inflammatory-active as well. The carotenoids representing the lipophilic non-enzymatic antioxidant system possess the tendency to accumulate and perform their protective activity mainly in cell membranes and lipoproteins by selectively quenching singlet oxygen and peroxyl radicals [6]. The carotenoids’ cells protection against oxidative stress and inflammation delivers a promising potential in reducing the risks of chronic degenerative and metabolic health conditions such as diabetes, photosensitivity disorders, obesity, aged-related eye diseases, several types of cancers and cardiovascular-related disorders [76,77,78]. Besides, certain carotenoids exert specific mechanisms in cancer chemoprevention and provitamin A activity that are antioxidant-independent properties. A summary of the studies related to health-promoting functional properties of different carotenoids samples of *Capsicum* is described in Table 2.

### 6.1. Antidiabetic Potential

The hypoglycaemic potential of *Capsicum*’s carotenoids has been reported in a few studies which showed interesting findings on the selective inhibitory activity of the lipophilic carotenoid fractions of different varieties and cultivars against α-amylase. The fractions from both immature and mature *C. chinense* cv. Habanero, which have been correlated to significant β-carotene bleaching antioxidant activity (*p* < 0.05; half maximal inhibitory concentration (IC_50_), 6.72 ± 0.09 and 21.7 ± 0.18 µg/mL, respectively), exhibited a significant α-amylase inhibitory activity (*p* < 0.01; IC_50_, 9.88 ± 0.4 and 29.6 ± 0.8 µg/mL, respectively) but were inactive α-glucosidase inhibitors [50]. The selective α-amylase inhibitory activity was also exhibited by those of *C. annuum*’s with IC_50_ values ranges between 6.9 and 28.6 µg/mL [79,80,81]. The inhibitory action on the hydrolyzing enzyme lengthens carbohydrate digestion time, resulting in a declined glucose absorption rate in the small intestine and a consequent decrease in post-prandial hyperglycaemia. A significant potential in preventing obesity-related insulin-resistance was exerted by non-acylated capsanthin-capsorubin-rich purified paprika pigments (PP). The fraction, which consisted of 44.3% capsanthin, 12.8% capsorubin, and capsanthin analog, showed a dose-dependent adipogenesis in mouse 3T3-L1 preadipocyte by increasing the activity of glycerol-3-phosphate dehydrogenase (GPDH) and acting as a good ligand for peroxisome proliferator-activated receptor gamma (PPARγ). The promotion of small adipocyte differentiation and adiponectin secretion by the cells are connected to the potential of PP in improving glucose tolerance [82]. PP is also able to attenuate inflammation in obesity-induced inflammatory adipocyte cells, which is closely associated with insulin resistance. The anti-inflammatory action is indicated by suppression of adipocytokine mRNA gene expression for inflammatory factors, such as interleukin-6 (IL-6), tumour necrosis factor-α (TNF-α), monocyte chemotactic protein-1 (MCP-1) and resistin, and reduction in nitric oxide release [82]. Moreover, capsanthin was suggested to have potential insulin sensitizing activity for its dose-dependent increased adiponection and phosphorylated adenosine monophosphate-activated protein kinase (p-AMPK) activities in high-fat, diet-induced obesity mouse models [83]. Zeaxanthin-rich *Capsicum* fruits can be a highly recommended diet for diabetic patients as zeaxanthin showed antidiabetic potential with auxiliary effects on related complications, including hypolipidemic and antidiabetic nephritic activities. A study on a diet-streptozotocin (STZ)-induced type 2 diabetic rat model showed that zeaxanthin at 200 and 400 mg/kg normalized the body weight and reduced fasting blood glucose by up to 24.7% and 34.7% (*p* < 0.05), respectively. The antidiabetic activities are also suggested to be associated with its modulation of lipid metabolism and antioxidative factors. In addition, zeaxanthin at 400 mg/kg provided renal protective effects in diabetic rats, as indicated by significantly lowered blood urea nitrogen (*p* < 0.05, 37.3%), urine levels of n-acetyl-β-d-glucosaminidase and albuminuria, and serum levels of inflammatory factors including TNF-α (*p* < 0.05; 12%), IL-2, IL-6 and the consequent nuclear factor kappa B (NFκB) (*p* < 0.01; 26.5%, 33.3% and 26.2%, respectively). Histopathological observations of ameliorated glomerular hypertrophy and thickening of the glomerular basement membrane, confirmed the antinephropathic effects of zeaxanthin [84].

### 6.2. Antiadipogenic and Anti-Obesity

The anti-obesity potential of capsanthin from red pepper has been studied in vitro in murine preadipocyte cell line 3T3-L1 adipogenesis model and pharmacologically validated in high-fat diet obesity mice. Capsanthin and a mixture of its esterified form possessed antiadipogenic activity with IC_50_ of 2.5 ± 0.45 µM and 12.5 ± 3.44 µM, respectively. The activity was superior to other carotenoids including lutein, zeaxanthin, β-carotene, β-cryptoxanthin and capsorubin (IC_50_ > 60 µM). Capsanthin also showed promising adrenoceptor-β_2_-agonistic activity with subsequent potent lipolytic and fatty acid burning activities. The associated excessive ATP production enhanced spontaneous locomotive activity with sustained weight loss in capsanthin-fed obese mice [83]. Although β-carotene and cryptoxanthin present at moderate amounts in pepper, both plasma carotenoids may contribute to lower risk of adiposity since their level was found to correlate significantly with overweight and obesity in children and adults. A significantly lower level of β-carotene and cryptoxanthin was observed in overweight and obese children compared to healthy weight children [93]. Meanwhile, an improvement of insulin-resistance in reducing adiposity was found to be associated with a high serum β-carotene concentration in prepubertal overweight boys [94].

### 6.3. Skin Photoprotective

Carotenoids have gained particular attention as protective agents in skin-photosensitivity-related disorders, namely erythema, photocarcinogenesis and photoageing [76]. Moreover, carotenoid-rich dietary antioxidants have been reviewed as potential systemic photoprotective agents towards skin damage induced by ultraviolet A (UVA) and ultraviolet B (UVB) radiations [95]. Capsanthin, capsorubin, β-carotene and lutein are the carotenoids of pepper that possess the ability to complement and support the dermal photoprotection system against UV radiation via their strong antioxidant defence, mainly as singlet oxygen quencher and peroxyl radicals scavenger [85,96]. Capsanthin and capsorubin showed a significant in vitro protective effect against UVB-induced cytotoxicity on normal human dermal fibroblast. At 1 µM, capsanthin and capsorubin showed decreasing cell viability (82% to 57%) in a dose-dependent manner to UVB exposure doses of 100 to 300 mJ cm^-2^, while lutein was only able to counteract the effect of the lowest dose. The photoprotection of the carotenoids significantly prevented UVB-induced DNA strand breaking and apoptotic cell death by decreasing the breaking formation and caspase-3 cleavage respectively, with the highest photoprotection exerted from capsorubin followed by capsanthin and lutein [85]. The efficacy of oral administration of red paprika-xanthophylls in suppressing UV-induced skin damage has been evaluated in a randomized, placebo-controlled, parallel group comparative clinical study involving Japanese males and females aged 30 to 50 years with skin phototype II. A daily intake of a capsule containing 9 mg total xanthophylls including 5 mg capsanthin and 0.5 mg β-cryptoxanthin, showed suppression of UV-induced erythema and pigmentation. The result was suggested to be associated with the strong singlet oxygen quencher properties of the xanthophylls which counter the UV-induced photooxidative stress and subsequent acute inflammation response [86]. Likewise, lutein and zeaxanthin in pepper may also have promising skin antioxidant photoprotection. The efficacy of lutein and zeaxanthin against skin lipid peroxidation and photoprotective activity was evaluated in a randomized, placebo controlled, 12-week clinical trial on forty healthy women (25–40 years of age) with signs of premature skin aging. The highest lipid peroxidation was exhibited through a combined oral (lutein 5 mg/zeaxanthin 0.3 mg) and topical (lutein 50 ppm/zeaxanthin 3 ppm) administration twice a day while a six-fold higher than placebo effect was exerted in photoprotective activity [97].

### 6.4. Macula Pigments

Lutein and zeaxanthin constitute the main components of macular pigment of the eye and are crucial in maintaining eye health. Both oxycarotenoid xanthophylls possess the ability to prevent and slow the progression of age-related macular degeneration (AMD) and cataracts. The xanthophylls act as protectors against light-induced oxidative damage, thereby maintaining the function and structure of the retina through their ability as blue light filters and excellent reactive oxygen species (ROS) scavenger [98,99]. Supplementation of lutein at 20 mg daily for 48 weeks has significantly increased the macular pigment optical density (MPOD), thereby leading to improvement in visual function in patients with early AMD and preventing its progression [98]. Furthermore, a clinical trial showed that lutein and zeaxanthin supplementation at 5.9 mg and 1.2 mg respectively, for six months has significantly increased their serum concentrations (*p* = 0.001 and 0.003, respectively), hence increasing the subjects’ central MPOD (0.25°: *p* = 0.001; 0.5°: *p* = 0.001) with no adverse clinical implications [100]. Higher intake of bioavailable lutein, zeaxanthin and some other carotenoids, like β-carotene, has also been associated with long-term reduced risk of advanced AMD [101]. Although advanced anti-angiogenesis therapy for AMD is currently available, fruit and vegetable diets rich in lutein and zeaxanthin are the recommended preventive strategy in reducing the incidence of AMD, due to the unaffordability of the therapy to all patients [102]. Green and orange pepper are among the best choices of lutein- and zeaxanthin-rich diets respectively, for the management of cataract and AMD, especially among the elderly, besides maintaining the eye health [32,56].

### 6.5. Antinociceptive/Analgesic and Anti-Inflammatory

Nociception, also easily explained as pain, and inflammation are mainly triggered by oxidative stress, under which conditions, pepper’s carotenoids may serve as one of the useful forms of therapeutic relief [3,87]. A carotenoids-rich fraction constituted mainly of β-carotene (344.0 ± 0.05 µg/g dry weight (DW)), β-cryptoxanthin (407.2 ± 0.05 µg/g DW) and violaxanthin (1671.0 ± 0.05 µg/g DW) from guajillo pepper of *C. annuum* possessed significant peripheral anti-nociceptive activity at 5, 20 and 80 mg/kg. Moreover, the fraction exhibited an induced central analgesic effect at 80 mg/kg with a more prolonged response time of pain reflex to thermal stimulus in mice compared with indomethacin (7 mg/kg). The pain withstood by the mice was suggested to be associated with the carotenoids’ free radicals scavenging and local prostaglandin inhibitory effects. Interestingly, the fraction also showed significant anti-inflammatory activity in carrageenan-induced mice paw oedema with a comparable response to that of indomethacin (7 mg/kg) at doses of 20 and 80 mg/kg [3]. The activity was also observed in carotenoid extract (2076 ± 10 μg/g dry weight) rich in capsanthin, lutein and β-carotene from Ukrainian cayenne bitter pepper of *C. annuum*. An ointment containing 0.2% of the extract exhibited good inhibitory effect against the formation and progression of inflammation in an adjuvant-induced oedema model. The effectiveness of the extract as a local anti-inflammatory agent is most probably due to their antioxidant mechanism which suppresses the alteration action of reactive oxygen intermediates in the area of inflammation [87].

### 6.6. Antihyperlipidemic and Cardioprotective

Peppers’ carotenoids are potential cardioprotective agents, as demonstrated by the inverse association in serum β-cryptoxanthin, lutein plus zeaxanthin, and plasma β-carotene levels with the prevalence of atherosclerosis and myocardial infarction in many epidemiological studies. Although some data are conflicting, there are supporting data for anti-inflammatory and antioxidant carotenoids on the chronic inflammation of the arterial wall and low-density lipoprotein cholesterol (LDL-C) oxidation respectively, which are closely linked to their preventive role against cardiovascular disease related to artherosclerosis [103,104,105]. Antioxidant xanthophylls possess the tendency to accumulate at the surface of lipoproteins and thus, improve the LDL-C resistance to oxidation, which prevent the pivotal steps of initiation and progression of the artherosclerosis while enhancing the high-density lipoprotein (HDL) function in removing excess cholesterol from the body [104]. Besides, red pepper also contains carotenoids as potential natural cholesterol metabolism regulators for prevention of atherosclerosis. A 1% red pepper supplementation to cholesterol-fed rabbits has significantly lowered plasma levels of triglyceride (TG), low density lipoprotein cholesterol (LDL-C), very low density lipoprotein cholesterol (VLDL-C), and very low density lipoprotein triglyceride (VLDL-TG), and atherogenic index (AI) (*p* < 0.05), whereas it ameliorated the high-density lipoprotein cholesterol (HDL-C) level (*p* < 0.05) during the experimental period of 12 weeks compared to the control group [88]. The plasma level of HDL-C is the lipid biomarker for assessing cardiovascular health for its strong inverse correlation with the risk of artherosclerotic cardiovascular disease [106]. Capsanthin showed the ability to increase the concentration of HDL-C in rat in a dose-dependent manner as revealed by the increment in apoA5 and lecithin-cholesterol acetyl transferase (LCAT) mRNA gene expression in rat liver, which links closely to HDL-C production [89]. HDL exerts its atheroprotective property via one of its mechanisms involving the function of LCAT in the reverse cholesterol transport process by creating a concentration gradient for the efflux of free cholesterol from peripheral cells to HDL particles to be excreted by the liver [106].

### 6.7. Hepatoprotective

The liver is the main storage site for carotenoids in the human body where the bioavailable β-cryptoxanthin, lutein, zeaxanthin and β-carotene of pepper may exert potential hepatoprotective effects. These dietary, anti-inflammatory-possessing antioxidants appear to be beneficial in the prevention and reduction of non-alcoholic fatty liver disease (NAFLD) [107,108]. The prevalence of NAFLD was found to be inversely correlated with the plasma lutein and zeaxanthin concentrations in Chinese adults [109]. Lutein showed remarkable protective effects in cholesterol-induced liver damages, such as hepatic steatosis in guinea pig and NAFLD in Sprague-Dawley rats, while zeaxanthin treatment produced lower hepatic lipid hydroperoxides and liver fibrosis in Mongolian gerbils with non-alcoholic steatohepatitis (NASH) [107]. Besides its antioxidant and anti-inflammatory properties, dietary β-cryptoxanthin showed novel actions in preventing or treating NAFLD through the regulation of macrophage polarization and liver homeostasis [108]. Meanwhile, β-carotene possessed a protective effect on liver damage and therapeutic potential on hepatic inflammation, fibrosis and cirrhosis [110]. These carotenoids also hold great potential in preventing and treating over-the-counter (OTC) analgesics-induced acute liver failure (ALF), as reviewed in Reference [62]. The ethanolic extract of red *C. annuum* fruit consisting mainly of capsanthin and capsoroubin was effective as a liver protectant against paracetamol-induced hepatotoxicity in male wistar albino rats. The extract produced a significant reduction in lipid profile (free fatty acids, phospholipids and triglycerides) and increased glycoprotein (*p* < 0.05) in liver tissue compared with the induced rats at doses of 250 and 500 mg/kg body weight [90].

### 6.8. Chemopreventive

The cancer-preventative promising potential of carotenoids is exerted partly through their role as natural anti-carcinogens owing to their excellent antioxidant and endogenous antioxidants enhancer effects. The anti-carcinogenesis property enables carotenoids to fight against carcinogen-induced oxidative damages that are involved in the multi-stage carcinogenesis [111,112]. Capsanthin, β-cryptoxanthin, lutein, zeaxanthin and β-carotene are the carotenoids of pepper that have been reviewed for their proven anti-carcinogenic effects with potential uses in cancer chemoprevention [112,113]. The mechanisms in counteracting tumour promotion include favouring antioxidant activity and protecting intercellular communications [114]. The carotenoids are anti-tumour promoters with the ability to inhibit in vitro 12-O-tetradecanoylphorbol-13-acetate (TPA)-induced Epstein-Bar virus early antigen (EBV-EA) activation and in vivo skin, colon and liver carcinogenesis in mice models [113]. The diesters of capsanthin and capsoroubin also showed potent in vitro anti-tumour promoter activity against TPA-induced EBV-EA activation. The activity of the major carotenoids, namely, capsanthin, capsanthin 3’-ester and capsanthin 3,3’-diester were proven to be potent in an in vivo two-stages carcinogenesis assay in mouse skin using 7, 12-dimethylbenz(a)-anthracene as an initiator and TPA as a promoter with a remarkable delay in papilloma formation and its number per mouse [115]. The chemopreventive activity of β-carotene, β-cryptoxanthin, lutein and zeaxanthin, as demonstrated by experimental models, could be correlated with their epidemiological, interventional and case-control studies, which mostly showed an inverse association between the dietary intake and the risk of certain cancers occurring in different tissues, including pancreatic, lung and skin cancers [76,112,116]. The antioxidant-active red paprika extract, capsanthin and β-carotene have been demonstrated to suppress the generation of ROS in hydrogen peroxide (H_2_O_2_)-treated WB-F344 rat liver epithelial cells, and blocked ROS from affecting the functional gap junction intercellular communication (GJIC) of the cells. The protective effects against H_2_O_2_-induced inhibition of GJIC was evidenced from the recovered connexin 43 (Cx43) mRNA expression, attenuated phosphorylation of Cx43 protein and mitogen-activated protein (MAP) kinases in pre-treated cells. The results suggested the chemopreventive potential of the extract and active carotenoids in the liver cells [91]. In addition, some carotenoids possessed anticancer potential as a resistance modifier by having the ability to inhibit the function of multidrug-resistance (MDR)-efflux proteins and eventually reverse the MDR of certain tumour cells. A study on the MDR-reversal effects of selected carotenoids (2 µg/mL) in human MDR-1 gene-transfected mouse lymphoma cell lines showed the effect at a decreasing order of capsoroubin > capsanthin > antheraxanthin > lutein > verapamil (5 µg/mL) > zeaxanthin. In addition, the carotenoids (2 µg/mL) also possessed the ability to induce apoptotic cell death. Antheraxanthin exhibited the highest rate of early apoptosis induction in drug-sensitive human breast cancer HTB-26 cells, whereas zeaxanthin and capsanthin showed the highest total apoptosis, comparable to that of the positive control, 12H-benzo(a)phenothiazine (M627, 50 µg/mL). An increase in death rate was observed in cells treated with violaxanthin and capsanthin [92].

### 6.9. Provitamin A Activity

Dietary provitamin A carotenoids are essential in those having inadequate supply of preformed vitamin A from its animal sources, or intolerant to the sources, especially among pre-school aged children and pregnant women [117,118]. Provitamin A carotenoids intake estimates approximately 35% of the total daily intake of vitamin A, with 30.4% β-carotene as the carotenoid of the highest intake [119]. Pepper fruit serves as one of the primary dietary sources of provitamin A carotenoids which helps to counter vitamin A deficiency (VAD) and consequently prevents abnormalities in growth, development, immune function and vision [120]. The provitamin A carotenoids of pepper include β-carotene, β-cryptoxanthin, α-carotene and cryptocapsin. β-Carotene stands out as the provitamin A with the highest activity (1 µg of retinol activity equivalent, RAE = 12 µg) compared with that of β-cryptoxanthin and α-carotene (1 RAE = 24 µg) [119,121]. Red-fruited *C. annuum* serves the best as a provitamin A source at the fully ripe stage, as the total provitamin A carotenoids of β-carotene and β-cryptoxanthin present at the highest level (838.80 International Units (IU) provitamin A/mg fresh weight) compared with at other maturity stages [122]. However, the fresh fruits are preferable as their all-trans carotenoids, the main form of vitamin A precursors, remain unaffected by oxidative degradation through processing and cooking [121]. Due to greater bioavailability of β-cryptoxanthin than β-carotene, the rich food sources of both carotenoids may contribute a considerably equivalent amount of vitamin A [121]. Structurally, a polyene chain with at least one terminal unsubstituted β-ionone ring and methyl groups at the correct number and position is the required characteristic in a carotenoid for being able to be converted into vitamin A via oxidative cleavage in the intestine [6]. Even at a high intake, the carotenoids remain as the safe form of vitamin A as their conversion is regulated by a feedback mechanism which prevents vitamin A overdose or toxicity [123]. Thus, the safe intake of vitamin A is recommended to constitute provitamin A carotenoids in combination with preformed vitamin A [119] to meet a daily requirement of 700 to 1300 RAE according to a person’s age and reproductive status [124].

## 7. Safety of *Capsicum* and Its Carotenoids

The adverse effects and toxicity of the whole pepper fruit have been reviewed and are mainly due to the “heat” principles, the capsaicinoids which include dermatological injury, opthalmic injury, nasal and respiratory toxicity, gastrointestinal problem and systemic toxicity [125]. Since capsaicinoids are accumulated predominantly in the capsaicin gland of the placenta and seed tissues, while carotenoids are concentrated in the sacrocarp of the fruit with a very low level of capsaicinoid (from 0.07 up to 80 mg/100 g fresh weight) [126], the unpleasant effects would be much less experienced when only the pericarp is utilised for the intended health benefits of its carotenoids. In addition, pepper is considered safe with no carcinogenic effects unless it is taken above normal human consumption and is aflatoxin contaminated, exceeding the acceptable limit [127]. A widely used natural dye, E 160c, is a specified paprika extract that contains principally capsanthin-capsorubin (>2.1%) as determinant for its red colour quality, and a very low content of capsaicin (<0.025%). It was re-evaluated to be safe as a food additive at the acceptable daily intake (ADI) of 24 mg/kg bw/day (1.7 mg carotenoids/kg bw/day) with no carcinogenicity or genotoxic potential [128]. No adverse effects were observed for daily intake of paprika oleoresin at 20 and 100 mg/kg for 12 weeks [47]. Meanwhile, hypercarotenemia increases of cancer risks and prooxidants-induced adverse effects of β-carotene, are more likely to be associated with overdoses of its supplementation rather than due to its high dietary intake [129,130].

## 8. Conclusions

Pepper serves as an excellent source of various types of lipid antioxidant carotenoids, particularly the mild type with less heat principles. Capsanthin, violaxanthin and zeaxanthin are available in abundance respectively, in the red, yellow and orange coloured fruits. The different carotenoids in mixtures available in pepper fruit provide a concerted protection against various chronic degenerative diseases, along with health promotion potentials in consequence to their multifaceted pharmacological effects. Pepper is highly recommended for its considerably safe and health-beneficial carotenoids, as humans are unable to synthesize them de novo and are dependent on their availability through diet.

## Figures and Tables

**Table 1:** Variable levels of main carotenoids in selected collections of sweet and hot *Capsicum.*

Species	Cultivar	Colour	Carotenoid Concentration									Reference
			1	2	3	4	5	6	7	8	9	
	Red Mountain	Red	31.8 ± 4.54	4.6 ± 0.71	2.73 ± 0.77	0.59 ± 0.16	ND	3.61 ± 0.44	ND	ND	43.32 ± 6.88	
	Magnifico	Red	28.33 ± 2.83	4.48 ± 0.78	2.06 ± 0.06	0.4 ± 0.04	ND	ND	ND	ND	35.99 ± 3.51	
	Nagano	Red	22.88 ± 1.65	4.01 ± 0.32	2.01 ± 0.05	0.31 ± 0.02	ND	ND	ND	ND	29.82 ± 2.01	
^a^ *C. annuum*	Preludium	Red	29.56 ± 0.67	2.67 ± 0.09	1.94 ± 0.17	0.33 ± 0.02	ND	ND	ND	ND	35.05 ± 0.90	[32]
	Adami Red	Red	28.62 ± 0.99	2.59 ± 0.17	1.88 ± 0.18	0.32 ± 0.02	ND	ND	ND	ND	33.94 ± 1.38	
	Raon Red	Red	13.26 ± 5.14	1.74 ± 0.82	0.83 ± 0.17	0.13 ± 0.07	ND	ND	ND	ND	13.51 ± 1.51	
	Red	Red	21.55 ± 5.93	3.09 ± 0.97	1.13 ± 0.21	0.30 ± 0.09	ND	ND	ND	ND	29.11 ± 1.52	
	RD-Glory	Red	3.98 ± 0.47	0.95 ± 0.09	0.86 ± 0.02	0.47 ± 0.24	ND	24.05 ± 0.16	ND	ND	37.25 ± 0.63	
	Mazzona	Orange	ND	ND	1.23 ± 0.01	1.12 ± 0.04	28.39 ± 1.00	151.39 ± 4.85	6.19 ± 0.09	0.29 ± 0.00	190.43 ± 5.66	
	Orange Glory	Orange	ND	ND	0.98 ± 0.09	1.09 ± 0.08	15.77 ± 1.52	145.92 ± 13.17	5.52 ± 2.00	0.26 ± 0.04	171.95 ± 17.13	
	Orange Star	Orange	ND	ND	0.62 ± 0.05	0.64 ± 0.08	25.27 ± 3.21	140.05 ± 14.48	4.47 ± 1.59	0.22 ± 0.10	172.77 ± 19.71	
^a^ *C. annuum*	Raon Orange	Orange	ND	ND	0.76 ± 0.01	0.55 ± 0.01	22.24 ± 0.51	88.80 ± 1.06	0.89 ± 0.07	ND	115.01 ± 1.46	[32]
	Mini Goggal Orange	Orange	ND	ND	0.63 ± 0.01	0.64 ± 0.01	17.32 ± 0.48	89.89 ± 2.89	1.87 ± 0.77	ND	111.83 ± 4.25	
	Orange	Orange	ND	ND	0.75 ± 0.03	0.61 ± 0.01	25.10 ± 0.39	115.53 ± 1.11	3.06 ± 0.32	ND	146.93 ± 1.93	
	OE-Glory	Orange	ND	ND	0.66 ± 0.16	0.48 ± 0.11	19.31 ± 4.00	85.06 ± 19.08	2.00 ± 0.49	0.28 ± 0.07	109.69 ± 24.32	
	Jorrit	Yellow	ND	ND	0.15 ± 0.04	0.03 ± 0.01	8.75 ± 2.29	0.66 ± 0.31	0.81 ± 0.25	0.14 ± 0.03	11.38 ± 3.14	
	Coletti	Yellow	ND	ND	0.21 ± 0.01	0.05 ± 0.00	13.83 ± 0.94	0.71 ± 0.06	1.48 ± 0.06	0.72 ± 0.30	18.2 ± 1.46	
	Sven	Yellow	ND	ND	0.15 ± 0.01	0.05 ± 0.01	13.16 ± 1.07	0.63 ± 0.04	1.06 ± 0.20	0.20 ± 0.01	16.21 ± 1.42	
^a^ *C. annuum*	Atalante	Yellow	ND	ND	0.17 ± 0.00	0.03 ± 0.00	11.97 ± 0.56	0.45 ± 0.09	ND	1.05 ± 0.57	15.31 ± 1.37	[32]
	Raon Yellow	Yellow	ND	ND	0.30 ± 0.00	0.43 ± 0.04	21.08 ± 0.84	2.22 ± 0.18	1.35 ± 0.41	2.85 ± 1.34	29.70 ± 0.67	
	Yellow	Yellow	ND	ND	0.29 ± 0.06	0.29 ± 0.00	18.32 ± 6.79	1.27 ± 0.08	ND	0.85 ± 0.09	22.32 ± 7.23	
	YW-Glory	Yellow	ND	ND	0.27 ± 0.03	0.27 ± 0.07	15.17 ± 1.25	1.19 ± 0.52	ND	0.55 ± 0.21	18.45 ± 1.02	
^b,c^ *C. annuum*	Pimenta PMO	Red	NA	NA	^b^ 4442.72 ± 1.0	^b^111.12 ± 0.19	^b^ 195.75 ± 0.25	^b^ 460.03 ± 3.13	NA	NA	^c^ 1064.35 ± 19.38	[44]
	Pimenta Amarela	Yellow	NA	NA	ND	ND	^b^ 312.79 ± 0.12	^b^ 8.78 ± 0.03	NA	NA	^c^91.26 ± 8.59	
^b,c^*C. baccatum* var. *umbilicatum*	Pimenta Chumbinho Baião	Red	NA	NA	^b^ 454.08 ± 0.20	^b^ 1456.24 ± 0.80	^b^ 139.85 ± 0.14	^b^ 1291.29 ± 0.40	NA	NA	^c^ 580.98 ± 51.91	[44]
	Pimenta Biquinho	Orange	NA	NA	ND	ND	^b^ 687.71 ± 0.66	^b^ 25.56 ± 0.02	NA	NA	^c^ 208.45 ± 12.65	
^b,c^ *C. chinense*	Pimenta Curuçazinho	Yellow	NA	NA	^b^ 33.48 ± 1.06	ND	^b^ 89.91 ± 0.65	^b^ 355.68 ± 0.35	NA	NA	^c^ 73.80 ± 2.93	[44]
	Pimenta Murupi	Yellow	NA	NA	ND	ND	^b^ 262.96 ± 0.08	^b^ 20.81 ± 5.99	NA	NA	^c^ 79.56 ± 7.12	

^a^ Concentration in mg/100 g dry weight; ^b^ Concentration in µg/100 g dry weight; ^c^ Concentration in µg/g dry weight; 1: Capsanthin; 2: Capsorubin; 3: β-Carotene; 4: β-Cryptoxanthin, 5: Lutein, 6: Zeaxanthin; 7: Antheraxanthin; 8: Violaxanthin; 9: Total carotenoids; ND: Not detected; NA: Not assessed.

**Table 2:** Summary of in vitro, in vivo and clinical data from studies related to health-promoting functional properties of extract, fractions and carotenoids from *Capsicum* sources.

Functional Property	Type of Study	Biological/Pharmacological/ Clinical Activity	Species/Variety/ Cultivar	Extract/Fraction/ Carotenoid	Carotenoid Content/Purity	Dose	Effects/Identified Mechanism	Reference
Hypoglycaemic	In vitro	α-Amylase inhibitory	*C. chinense* cv. Habanero	Lipophilic hexane fraction from ethanol extract of immature (I) and mature (M) fruits.	62.7 ± 5.5 (I) and 362 ± 7.8 (M) mg β-carotene eq./100 mg FW	IC_50_, 9.88 (I) and 29.58 (M) µg/mL	A selective α-amylase inhibitory activity. No α-glucosidase inhibitory activity.	[50]
			*C. annuum* var. *acuminatum* small	Lipophilic hexane fraction of ethanol extract from deseeded air-dried mature fruits.	Not determined	IC_50_, 6.9 µg/mL	A selective α-amylase inhibitory activity. Inactive as α-glucosidase inhibitor.	[79]
			*C*. *annuum* var. *cerasiferum*	Lipophilic hexane fraction of ethanol extract from deseeded air-dried mature fruits.	Not determined	IC_50_, 20.1 µg/mL	A selective α-amylase inhibitory activity. No α-glucosidase inhibitory activity.	[79]
			*C*. *annuum* var. *acuminatum* big	Lipophilic fraction of ethanol extract from deseeded air-dried immature fruits.	Not determined	IC_50_, 8.7 µg/mL	A selective α-amylase inhibitory activity. Inactive as α-glucosidase inhibitor.	[80]
			*C. annuum* cv. Fiesta, Acuminatum, Orange Thai and Cayenne Golden	Lipophilic hexane fraction of ethanol extract from deseeded air-dried immature (I) fruits.	Not determined	IC_50_, ranged from 9.1 to 28.6 µg/mL (I)	A selective α-amylase inhibitory activity, Fiesta > Cayenne Golden > Acuminatum > Orange Thai. No α-glucosidase inhibitory activity.	[81]
Anti-obesity	In vitro	Antiadipogenic on murine 3T3-L1 pre-adipocytes	Not determined	Capsanthin purified from commercialised red pepper powder.	Capsanthin, 100%	IC_50_, 2.5 ± 0.45 µM	The activities resulted from potent adrenoceptor-β2-agonistic which is linked to the activation of hormone sensitive lipase.	[83]
		Lipolytic in differentiated 3T3-L1 adipocytes				ED_50_, 0.872 ± 0.06 µM		
	In vivo	Inhibition of weight gain in high-fat-diet-induced obese female C57BL/6C mice	Not determined	Capsanthin purified from commercialised red pepper powder.	Capsanthin, 100%	1, 5, and 10 µmol	A dose-dependent enhancement of locomotive activity associated with excessive production of ATP with progressive weight loss.	[83]
Skin photoprotective	In vitro	Anti UVB-induced cytotoxicity on normal human dermal fibroblasts	Not determined	Purified capsanthin, capsorubin and lutein from commercialised paprika oleoresin.	Capsanthin, 100% Capsorubin, 100% Lutein, 100%	1 µM	Protective effects by producing significant decrease in the formation of UVB-induced DNA strand break and counteracting caspase-3 cleavage.	[85]
	Clinical	Anti-UV-induced skin damage in a double-blind placebo-controlled study (Japanese male and female, aged 30 to 50 years with skin phototype II).	Not determined	A commercial paprika-xanthophyll preparation (PapriX- oil).	333 mg of PapriX-oil gelatine capsule (l 9 mg total xanthophylls, 5 mg capsanthin, and 0.5 mg cryptoxanthin)	One capsule orally with a meal every evening for 5 weeks.	Suppression of UV-induced erythema and pigmentation by the xanthophylls’ strong singlet oxygen quenching activity that counteracted UV-induced photooxidative stress and acute inflammation response.	[86]
Anti-Inflammatory	In vitro	Anti-inflammatory in obesity-induced inflammation in 3T3-L1 adipocytes co-cultured with RAW264.7 macrophages.	Not determined	Purified paprika pigments from commercialised paprika carotenoids.	Capsanthin, 44.3% Capsorubin, 12.8% Capsanthin analog	15, 30, 60 µg/mL	Attenuation of inflammation in the 3T3-L1 adipocytes by dose dependant suppression of adipocytokine mRNA gene expression for IL-6, TNF-α, MCP-1 and resistin, and significant (*p* < 0.05) deceased versus control in nitric oxide release.	[82]
	In vivo	Anti-inflammatory in carrageenan-induced mice paw oedema.	*C*. *annuum* var. *guajillo* (Guajilo 15660)	Petroleum ether fraction of acetone extract from dried fruits.	β-carotene, 10.01% β-cryptoxanthin, 11.96% Violaxanthin, 49.06%	5, 20 and 80 mg/kg	Significant (*p* < 0.05) reduction of oedema at 5 hr time point, comparable to indomethacine (7 mg/kg)	[3]
		Anti-inflammatory in adjuvant-induced mice paw oedema.	*C*. *annuum* (Ukrainian cayenne bitter pepper)	Petroleum ether fraction of acetone extract from air-dried fruits.	Carotenoid extract containing 69.3% yellow and 30.7% red fractions rich in capsanthin, lutein and β-carotene.	Topical application of ointment containing 2 mg extract/g daily for 20 days.	Reduction of serum cholinesterase activity by 1.3 times and double decrease in the serum seromucoid concentration that indicated good inhibitory activity.	[87]
Anti-Hyperlipidemic	In vivo	Inhibition of CETP activity and anti-atherosclerotic in cholesterol-fed male New Zealand white rabbits.	Not determined	Mixture of red pepper powder, Purina lab Chow (Purina Chemical, Korea) and 1% cholesterol.	Red pepper powder, 1%	100 g supplement/day for 12 weeks	Significant (*p* < 0.05) lowered in total cholesterol, triglyceride, LDL-C, VLDL-C, and VLDL-TG levels, atherogenic index and CETP activity whereas higher HDL-C level than in the control group during the experimental period.	[88]
		Promotion of plasma LDL-C levels and hepatic gene expression in young male Wistar rats.	Not determined	Purified capsanthin from non-acylated capsanthin powder.	Capsanthin, 100%	0·25 and 0·49 mmol/kg diet ad libitum for 2 weeks	A dose-dependent increment of HDL-C associated with up-regulation of mRNA for apoA5 and LCAT.	[89]
Hepatoprotective	In vivo	Liver protectant against paracetamol induced hepatotoxicity in male albino Wistar rats.	*C*. *annuum*	Ethanol extract of dried red fruit powder.	Lycopene, 4.69 ± 0.01 mg/g tissue	250 and 500 mg/kg body weight	Improvement of liver functions as indicated by significant decreased in liver weight and lipid levels while increased in glycogen and glycoprotein in liver tissue as well as decreased in serum liver markers (AST, ALT ALP) and bilirubin, (*p* < 0.001) versus untreated rats.	[90]
Chemopreventive	In vitro	Protective activity against H_2_O_2_-induced inhibition of GJIC in WB-F344 rat liver epithelial cells.	*C. annuum*	Diethyl ether fraction of the red fruits (RPE), capsanthin (CST) and β-carotene (BCT).	RPE CST, 100% BCT, 100%	RPE, 10 and 25 µg/mL CST, 0.5 and 1.0 µM BCT, 0.1 and 0.5 µM	RPE, CST and BCT prevented GJIC inhibition by blocking the generation and actions of ROS and enhancing Cx43 mRNA expression, protein levels and the activity of ERK1/2, p38 and JNK MAP kinases.	[91]
		MDR-efflux protein inhibitory in human MDR-1 gene-transfected L1210 mouse lymphoma cells and human breast cancer cells MDA-MB-231 (HTB-26).	*C. annuum*	Purified capsanthin and capsorubin from red paprika.	Capsanthin, 100% Capsorubin, 100%	4 µg/mL	Capsanthin and capsorubin enhanced rhodamine 123 accumulation 30-fold relative to nontreated lymphoma cells that suggested their MDR reversal effect on the cells.	[92]
		Apoptosis induction in human breast cancer cells MDA- MB-231 (HTB-26).	*C. annuum*	Purified capsanthin from red paprika.	Capsanthin, 100%	2 µg/mL	Capsanthin induced apoptosis in both tumour cells comparable to that of the positive control, M627 (50 µg/mL).	[92]

β-Carotene eq./100 mg FW: β-Carotene equivalent/100 mg fresh weight; IC_50_: Half maximal inhibitory concentration. UV: Ultraviolet; UVB: Ultraviolet B; ED_50_: Effective dose 50; ATP: Adenosine triphosphate. mRNA: messenger ribonucleic acid; IL-6: Interleukin-6; TNF-α: Tumour necrosis factor α; MCP-1: Monocyte chemotactic protein-1; CETP: Cholesteryl ester transfer protein; VLDL-C: Very low density lipoprotein chlolesterol; VLDL-TG: Very low density lipoprotein triglyceride; HDL-C: High density lipoprotein cholesterol; LDL-C: Low density lipoprotein cholesterol; LCAT: Lecithin cholesterol acyltransferase. AST: Aspartate aminotransferase; ALT: Alanine aminotransferase; ALP: Alkaline phosphatase; GJIC: Gap junction intercellular communication; ROS: Reactive oxygen species; ERK1/2: Extracellular signal-regulated kinases 1 and 2; JNK MAP: Jun amino-terminal mitogen-activated protein; MDR: Multidrug resistance; MDA-MB-231: M.D. Anderson metastatis breast cancer cell line; M627: 12H-benzo(a)phenothiazine.
